# Incidence of constitutive and inducible clindamycin resistance among hospital-associated *Staphylococcus aureus*

**DOI:** 10.1007/s13205-013-0133-5

**Published:** 2013-04-02

**Authors:** B. Sasirekha, M. S. Usha, J. A. Amruta, S. Ankit, N. Brinda, R. Divya

**Affiliations:** Department of Microbiology, Center for Post Graduate Studies, Jain University, 18/3, 9th Main, Jayanagar 3rd Block, Bangalore, 560011 India

**Keywords:** Inducible clindamycin, D test, *Staphylococcus aureus*, Inducible MLS_B_ phenotype

## Abstract

Clindamycin is one of the important alternative antibiotics in the therapy of *Staphylococcus aureus* infections. Clinical failure of clindamycin therapy has been reported due to multiple mechanisms that confer resistance to macrolides, lincosamides and Streptogramin B (MLS_B_) antibiotics. In vitro routine tests for clindamycin susceptibility may fail to detect inducible clindamycin resistance due to *erm* genes, resulting in the treatment failure. Although data from the developed countries have shown to be enormity of the problem, data from the developing countries are lacking. The aim of the study was to distinguish different resistance phenotypes in erythromycin-resistant *S. aureus* by a simple double-disc diffusion test (D test). A total of 153 *S. aureus* isolates were subjected to routine antibiotic susceptibility testing, including cefoxitin disc (30 μg) and by oxacillin screen agar. Inducible clindamycin resistance was tested by ‘D test’ as per CLSI guidelines. Odds ratios (OR) and 95 % confidence intervals (95 % CI) were calculated. *P* values were calculated using SPSS (version 18). Among 153 *S. aureus* isolates, 42 (27.45 %) were resistant to methicillin, whereas 111 (72.54 %) were methicillin susceptible. Out of the 63 (41.17 %) erythromycin-resistant *S. aureus* isolates, 14 (9.15 %) showed inducible resistance [*P* = 0.0002, odds ratio (OR) 18.30; 95 % confidence interval (CI) 8.72–25.77), 20 (13.07 %)] showed constitutive resistance (*P* = 0.002, OR 14.38, 95 % CI 5.33–21.49), while the remaining 29 (18.95 %) showed inducible phenotype. Inducible and constitutive resistance was found to be higher in MSSA when compared with MRSA. Clinical laboratories should perform D test routinely to guide the clinicians about the inducible clindamycin resistance and to prevent misuse of antibiotics.

## Introduction

*Staphylococcus aureus* (*S. aureus*) is a leading cause of nosocomial and community-acquired infections in every region of world. The increasing prevalence of methicillin resistance among *Staphylococci* is an increasing problem (Yilmaz et al. 2007). This has led to the renewed interest in the usage of macrolide–lincosamide–streptogramin B (MLS_B_) antibiotics to treat *S. aureus* infections with clindamycin being the preferred agent due to its excellent pharmacokinetic properties (Deotale et al. 2010). However, the wide spread use of MLS_B_ antibiotics has led to an increase in the number of *Staphylococcal* strains acquiring resistance to MLS_B_ antibiotics (Ajantha et al. 2008).

Resistance to MLS_B_ can occur by two different mechanisms: an active efflux mechanism encoded by the *msrA* gene (macrolides Streptogramin B resistance) and ribosomal target modification encoded by the *erm* gene (MLS_B_ resistance) (Leclercq 2002). The expression of the MLS_B_ phenotype can be constitutive or inducible in the presence of low levels of inducers, such as erythromycin. *erm* genes encode enzymes that confer inducible or constitutive resistance to MLS agents via methylation of the 23S ribosomal RNA, thereby reducing binding by MLS agents to the ribosome (Fiebelkorn et al. 2003). In vitro, *S. aureus* isolates with constitutive resistance are resistant to erythromycin and clindamycin, and isolates with inducible resistance are resistant to erythromycin, but appear susceptible to clindamycin. In vivo, therapy with clindamycin may select for constitutive *erm* mutants (Leclercq 2002) that may lead to clinical failure (Siberry et al. 2003).

iMLS_B_ (inducible clindamycin) is not recognized using standard susceptibility test methods, including standard broth-based or agar dilution susceptibility tests (Fiebelkorn et al. 2003), including the Vitek system (Schreckenberger et al. 2004) and disc diffusion testing with erythromycin (E) and clindamycin (CL) discs (double discs) in nonadjacent positions (Gadepalli et al. 2006). Further reports on inducible clindamycin resistance are scanty in India. Therefore the present study was undertaken to determine the incidence of MLS_B_ resistance in the clinical isolates of *S. aureus* and to study the antibiotic sensitivity pattern of *S. aureus* isolates having the iMLS_B_ phenotype.

## Materials and methods

This prospective study was conducted for a period of 7 months from July 2010 to January 2011. A total of 153 *Staphylococcal* isolates were recovered from various clinical samples at the Microbiology Laboratory of Sri Bhagawan Mahaveer Jain hospital, a tertiary care hospital in Bangalore. Duplicate isolates from the same patient were not included in the study. Of 153 *S. aureus* isolates, 110 (71.89 %) were recovered from pus, 13 (8.49 %) from sputum, 11 (7.18 %) from ear swab, 9 (5.88 %) from blood, 5 (3.26 %) from urine and 5 (3.26 %) from tissue bits.

Isolates were identified up to species level by conventional methods (Gram stain, growth on mannitol salt agar, slide and tube coagulase test, DNase test and by biochemical test) (Kloos and Banerman 1999).

### Antibiotic susceptibility testing

The isolates were subjected to susceptibility testing by Kirby Bauer disc diffusion method on Mueller–Hinton agar plates using erythromycin (15 μg), clindamycin (2 μg), mupirocin (5 μg), fusidic acid (30 μg), pristinomycin (15 μg), linezolid (30 μg), vancomycin (30 μg), teicoplanin (30 μg), rifampicin (5 μg), chloramphenicol (30 μg), co-trimoxazole (30 μg), ciprofloxacin (5 μg), gentamicin (30 μg), amikacin (30 μg), and tetracycline (30 μg). The results were interpreted as per Clinical Laboratory Standards Institute (CLSI) guidelines (Clinical and Laboratory Standards Institute 2009) methicillin resistance was detected by cefoxitin disc diffusion method and by oxacillin screen agar (5 % NaCl, 6 μg/ml oxacillin).

### D-Test

Isolates that were erythromycin resistant was tested for inducible resistance by the ‘D test’ as per CLSI guidelines. Erythromycin (15 μg) disc was placed at a distance of 15 mm (edge to edge) from clindamycin (2 μg) on Mueller–Hinton agar plates previously inoculated with 0.5 McFarland bacterial suspensions. Plates were analyzed after 18 h of incubation at 37 °C. Interpretation of the inhibition zone diameters was as follows: If an isolate was erythromycin resistant and clindamycin susceptible, with a D-shaped inhibition zone around the clindamycin disc, it was considered to be positive for inducible resistance (D test positive, iMLS_B_ phenotype). If the isolate was erythromycin resistant and clindamycin susceptible, with both zones of inhibition showing a circular shape, the isolate was considered to be negative for inducible resistance (D test negative, MS phenotype), but to have an active efflux pump. If the isolate was erythromycin resistant and clindamycin resistant, the isolate was considered to have the macrolide–lincosamide–Streptogramin B constitutive (cMLS_B_ phenotype) (Steward et al. 2005). The quality control of the erythromycin and clindamycin disc was performed with *S. aureus* ATCC 25923.

### Statistical analysis

Demographic data were collected and SPSS version 18 was used for all statistical analysis. Odds ratios (OR) and 95 % confidence intervals (95 % CI) were calculated. *P* < 0.05 was considered statistically significant.

## Results

Of the 153 *S. aureus* isolates, 42 (27.45 %) were resistant to methicillin-resistant *S. aureus* (MRSA) and 111 (72.54 %) were methicillin-susceptible *S. aureus* (MSSA). Of the total 153 *S. aureus* isolates, 144 isolates (94.11 %) were coagulase positive and 9 (5.88 %) were coagulase negative. A total of 38 of the coagulase-positive *S. aureus* isolates showed methicillin resistant and four coagulase-negative isolates showed methicillin resistance.

Of the total 42 MRSA, 32 (76.19 %) isolates belonged to male and 10 (23.80 %) to female patients. Our study showed the highest percentage of MRSA occurrence in patients with the age group of 20–30 years. The number of MRSA isolates was significantly different among various age groups (*P* < 0.0001).

**Fig. 1:**
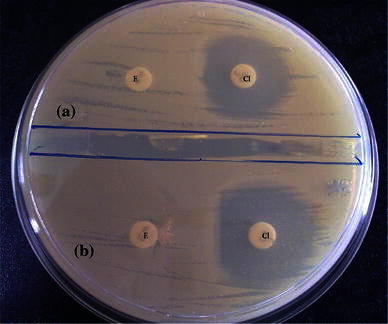
Disc diffusion test for inducible clindamycin resistance. **a** Circular zone of inhibition around clindamycin suggestive of MS phenotype. **b** D-shaped zone of inhibition around clindamycin suggestive of inducible MLS_B_ phenotype

Out of the 153 *S. aureus* isolates, 63 (41.17 %) of them were erythromycin resistant. These isolates when subjected to D test, 20 (13.07 %) isolates showed resistant to erythromycin and clindamycin indicating constitutive MLS_B_ phenotype. Out of the 43 isolates that showed clindamycin sensitivity, 14 (9.15 %) isolates showed positive D test indicating inducible MLS_B_ phenotype, while 29 (18.95 %) showed true sensitivity to clindamycin (D test negative indicating MS phenotype). 58.82 % had the susceptible phenotype (E-S, CL-S) (Table 1; Fig. 1).

**Table 1:** Susceptibility to erythromycin and clindamycin among all *Staphylococcal* isolates

Phenotypes	MRSA (%)	MSSA (%)	Total (%)
E-S, CL-S	26 (16.99)	64 (41.83)	90 (58.82)
E-R, CL-R (constitutive MLS_B_)	8 (5.22)	12 (7.84)	20 (13.07)
E-R, CL-S, D test positive (inducible MLS_B_)	1 (0.65)	13 (8.49)	14 (9.15)
E-R, CL-S, D test negative (MS)	9 (5.88)	20 (13.07)	29 (18.95)
Total	42 (28.74)	111 (71.23)	153

*E* erythromycin, *CL* clindamycin, *S* sensitive, *R* resistant, *constitutive MLS*_*B*_ constitutive resistance to clindamycin, *inducible MLS*_*B*_ inducible resistance to clindamycin, *MS* ms phenotype

Constitutive MLS_B_ phenotype was 5.22 % and the inducible MLS_B_ phenotype was 0.65 % in MRSA, while in methicillin sensitive *Staphylococcal* isolates, the constitutive MLS_B_ phenotype was 7.84 % and the inducible MLS_B_ phenotype was 8.49 %.

The E-S and CL-S phenotype predominated over the inducible resistance phenotype and constitutive resistance phenotype among MRSA and MSSA isolates. The percentage of inducible and constitutive resistance was higher amongst MSSA isolates when compared with MRSA isolates. Out of the 14 iMLS_B_ phenotype *S. aureus* isolates, 11 (78.57 %) isolates were isolated from pus, followed by 2 (14.28 %) isolates that were isolated from ear swab and 1 (7.14 %) isolate from tissue bits. iMLS_B_ phenotype was found more in males (71.42 %).

When the results were statistically compared between methicillin-sensitive *Staphylococcal* isolates, the constitutive CL-R phenotype was determined to be 1.5 times greater (*P* = 0.002, OR 14.38, 95 % CI 5.33–21.49) and the inducible resistance phenotype 13 times greater (*P* = 0.0002, OR 18.30, 95 % CI 8.72–25.77) than that in methicillin-resistant *Staphylococcal* isolates.

The susceptibility of iMLS_B_ phenotypes isolated were amikacin 85.71 %, gentamicin 78.57 %, ciprofloxacin 50 %, cotrimoxazole 57.14 % and tetracycline 85.71 %. iMLS_B_ phenotype *S. aureus* isolates were 100 % sensitive to vancomycin, teicoplanin, mupirocin, fusidic acid and linezolid, respectively.

## Discussion

The increasing frequency of *Staphylococcal* infections among patients and changing patterns in antimicrobial resistance have led to renewed interest in the use of clindamycin therapy to treat such infections (Frank et al. 2002). Clindamycin is frequently used to treat skin and bone infections because of its tolerability, cost, oral form and excellent tissue penetration, and the fact that it accumulates in abscesses and no renal dosing adjustments are needed (Kasten 1999). Good oral absorption makes it an important option in outpatient therapy or as follow-up after intravenous therapy. Clindamycin is a good alternative for the treatment of both methicillin-resistant and susceptible *Staphylococcal* infections (Fiebelkorn et al. 2003).

The very high rates of methicillin resistance among *S. aureus* isolates have been noted in developed countries; especially, in Western Pacific regions both in community-acquired and nosocomial infections (Diekema et al. 2001). In West Asia, MRSA prevalence ranged from 12 to 49.4 % in six different hospitals of Saudi Arabia (Hussain et al. 2008; Baddour et al. 2006). In European countries, MRSA rates varied from 0.6 % in Sweden to 40.2–45 % in Belgium, Greece, Ireland, Italy, UK, and Israel (Blandino et al. 2004; Sader et al. 2006). In a study performed in 17 different regions of Russia, methicillin resistance among *S. aureus* strains was between 0 and 89.5 %. In our study, methicillin resistance *S. aureus* was found to be 27.45 %. Similar prevalence rate of MRSA was obtained from other workers in India—22.8 % by Pal and Ayyagari (1991), 26.9 % by Shittu and Lin (2006) and 26.6 % Mehta et al. (2007). Although lesser and higher percentage was obtained by other workers—2.4 % Pulimood et al. (1996), 54.85 by Dar et al. (2006) and 65 % by Borg et al. (2006). The differences in the prevalence of MRSA among different countries and between different regions in a country could be due to difference in the study design, population and geographical distribution and the variation is probably due to differential clonal expansion and drug pressure in community. Further, it emphasizes the importance of local surveillance in generating relevant local resistance data that can guide empiric therapy.

In our study, there was no isolate with reduced susceptibility to glycopeptides, and all isolates were found susceptible to vancomycin, teicoplanin, fusidic acid, mupirocin and linezolid. Rahbar and Hajia (2007) also found all iMLS_B_ isolates susceptible to linezolid and vancomycin. Disc diffusion test for vancomycin in *Staphylococci* is no longer recommended by CLSI starting from 2009 and that the ability of teicoplanin disc diffusion test to differentiate resistant from susceptible strains is not known (Clinical and Laboratory Standards Institute 2009).

The incidence of MLS_B_ resistance varies significantly by geographical region. In our study, the percentage of inducible resistance and MS phenotype was higher amongst MSSA (8.49 and 13.07 %) when compared with MRSA (0.65 and 5.88 %). This was in concordance with a few of the studies reported before. Schreckenberger et al. (2004) and Levin et al. (2005) showed higher percentage of inducible resitance in MSSA (19–20 %) as compared to MRSA (7–12 %), 12.5 % MRSA and 68 % MSSA, respectively. The true incidence of the MLS_B_ phenotype of *S. aureus* depends on the patient population studied, and the geographical region, the hospital characteristics and methicillin susceptibility (MRSA or MSSA).

In our study, 8.49 % MSSA isolates were of the iMLS_B_ phenotype, which is in concordance with other workers, who have found that 4–15 % of their MSSA isolates were of the iMLS_B_ phenotype (Yilmaz et al. 2007). O’Sullivan et al. (2006) reported that a 15-mm distance, in an edge to edge position, had a 100 % sensitivity and specificity, while the 22-mm distance, in an edge to edge position, had a sensitivity of 87 % and a specificity of 100 % when compared with the presence of the *erm* gene as the gold standard for the detection of inducible CL resistance. Further Ajantha et al. (2008) in their study identified additional 14 % iMLS_B_ strains with 15 mm edge to edge interdisc distance. In the present study, we have maintained the narrow disc range to reduce the error rate of identifying iMLS_B_ isolates.

In the present study, 13.07 % of erythromycin-resistant *Staphylococcal* isolates showed true clindamycin susceptibility. Patients with infections caused by such isolates can be treated with clindamycin without emergence of resistance during therapy. In the current study, iMLS_B_ phenotype was found more in males (71.42 %). Male patient predominance most likely due to the fact that exposure is greater.

In the light of the restricted range of antibiotics available for the treatment of methicillin-resistant *Staphylococcal* infections and the known limitations of vancomycin, clindamycin should be considered for the management of serious soft tissue infections. Further, using clindamycin use of vancomycin can be avoided (Gupta et al. 2009). In addition, such testing can provide information about resistant to MLS phenotype group of antibiotics and can be useful for surveillance studies related to MLS resistance in *Staphylococci*.
